# The Relationship between Mortality of Lung Transplant Recipients and Serum Cyclosporine Levels: Joint Modeling of Time-to-Event Data and Longitudinal Data

**Published:** 2017-08-01

**Authors:** F. S. Hosseini-Baharanchi, E. Hajizadeh, A. R. Baghestani, K. Najafzadeh

**Affiliations:** 1 Minimally Invasive Surgery Research Center (MISRC), Iran University of Medical Sciences, Tehran, Iran; 2 Department of Biostatistics, Faculty of Medical Sciences, Tarbiat Modares University, Tehran, Iran; 3 Department of Biostatistics, Faculty of Paramedical Sciences, Shahid Beheshti University of Medical Sciences, Tehran, Iran; 4 Director of Organ Transplantation, Ministry of Health of Iran; 5 Lung Transplant Research Center, Masih Daneshvari Hospital, National Research Institute of Tuberculosis and Lung Diseases, Tehran, Iran

**Keywords:** Lung transplantation, Graft rejection, Cyclosporine, Mortality, Survival, Models, statistical, Lung diseases

## Abstract

**Background:**

Lung transplantation (LTx) is a well-accepted treatment that can prolong survival of patients with advanced lung disease.

**Objective:**

To evaluate the association between serum cyclosporine level (SCL) pattern and mortality of LTx recipients.

**Methods:**

This retrospective cohort study included 1019 observations on 38 patients who underwent LTx in Masih Daneshvari Hospital from 2000 to 2013. The analysis applied a joint model with shared random effects.

**Results:**

The mean±SD age of the recipients was 36±14.5 years. The findings indicated that sex, age, body mass index (BMI), the underlying disease, and cytomegalovirus infection were not associated with mortality. The mortality risk for the recipients with acute rejection (AR) history was 1.54 times that of the recipients who had none (95% CI: 1.08–2.19). The association parameter in the joint model (α = 0.8) showed that higher SCL was associated with lower mortality risk (95% CI: 1–1.011). A slightly linear decreasing trend in SCL mean was found after 10 months post-LTx; a significant 2% per month (95% CI: -0.03 to -0.019).

**Conclusion:**

AR history was found to be a risk factor in mortality in Iranian LTx recipients. Given the association between the higher SCL and lower mortality found in this study, it is recommended to pay serious attention to SCL changes in the overall post-transplantation survival assessment in Iranian LTx recipients.

## INTRODUCTION

Lung transplantation (LTx) is a treatment for patients with end-stage lung disease [1]. Survival rates were 79% and 53% at one and five years, respectively, according to ISHLT report. Low and high age, underlying disease, and rejection within the first year after LTx were significant risk factors for death [2]. Reported survival rates were 67% and 40% at one and five years, respectively, in Iran. Furthermore, underlying disease was a significant factor of the recipient’s median lifespan [3]. At least one episode of acute rejection (AR) within the first year was reported in 35% of adult lung recipients, with 89% of these being treated between 2004 and 2010 [2]. Between 2004 and 2010, those on a cyclosporine-based regimen had the highest reported ARs within the first year. AR rates are categorized based on the use of induction therapy and a maintenance immunosuppression regimen. The highest rejection rates were reported for the combination of cyclosporine and azathioprine therapy [2].

Cyclosporine, as a maintenance immunosuppressive regimen, has been introduced in solid organ transplantation and revolutionized this field [4]. However, therapeutic drug monitoring of cyclosporine to optimize efficacy and safety is still of clinical interest. No consensus has yet been attained on the criteria needed to increase immunosuppressive efficacy while limiting the side effects of cyclosporine. No relationship could be found between administered doses and clinical effects; fixed doses of cyclosporine is not the best way to use the drug. Therefore, the monitoring of the serum cyclosporine level (SCL) is mandatory to modify the individual doses of the drug to avoid side-effects. In addition, the correlation between SCL and clinical outcomes has been investigated in kidney transplant recipients [5]. 

Cyclosporine is one of the most commonly-used immunosuppressive drugs for Iranian recipients. It was observed that 2-hour post-dose cyclosporine was a predictor of long-term graft survival in kidney transplantation in the first six months post-transplantation [6]. The therapeutic drug monitoring of the SCL has been established as part of the routine clinical treatment for patients after organ transplantation [4]. According to the LTx manual, the optimal SCLs are 250–300 ng/mL in 1–3 months, 250–300 in 3-6 months, 200–250 in 6-12 months, and 150-250 ng/mL after one year. The target ranges for recipients older than 55 years or for those with cystic fibrosis and Burkholderia cepacia are 250–300, 250–300, 200–250, and 150–250 ng/mL, respectively [7]. 

The main objective of this study was to characterize the association between SCL and mortality controlling for other acute factors in Iranian LTx recipients. The SCL is a longitudinal variable that could be taken into account using longitudinal models. Recently, the joint modeling of two outcomes has been proposed especially when one is a time-to-event outcome and the other is a longitudinal outcome [8]. This methodology considers a sub-model for each outcome and relates them through random effects, providing the most valid and efficient inferences. A joint model was applied in this study to characterize the relationship between SCL and mortality. Finding the significant risk factors associated with mortality was the other objective of this study.

## MATERIALS AND METHODS

Study Population

We conducted a retrospective cohort study on 38 LTx recipients at Masih Daneshvari Hospital (National Research Institute of Tuberculosis and Lung Diseases), affiliated to Shahid Beheshti University of Medical Sciences, Tehran, Iran, from 2000 to 2013. There were 71 LTx recipients in this center during this period, 30 of whom died within the first two weeks post-LTx. Three recipients with missing SCLs were also excluded from the study. 

The primary outcome variable was time from LTx to death or the end of the study. The recipient’s fasting SCL, which was measured over the study period, was the other outcome of interest. The date of LTx and follow-up visits were known. Patients were followed up on weekly, for three months after LTx, and monthly from the third month to the first year. Subsequent follow-ups were performed whenever recipients had complications or undesirable changes in the SCL or received drugs that may have interacted with the SCL. The measuring date and SCL for every recipient in each visit from ICU to the latest follow-up visit were known. Seven recipients were switched to tacrolimus: 1 with severe neuropathy, 2 with diabetic ketoacidosis, and 4 with progressive onset of chronic rejection. We used their serum immunosuppressive level information until starting cyclosporine tapering. In this study, the occurrence of AR grade 1 and higher according to pathological findings was included as a binary variable; occurrence of at least one episode vs. none. Classification of AR based on the intensity of the infiltrates (graded in the following manner grade A0 [none], A1 [minimal], A2 [mild], A3 [moderate], and A4 [severe]) was performed according to ISHLT [9]. Since almost all the donors in Iran were infected with cytomegalovirus (CMV), all recipients received ganciclovir or valcyte up to three months after LTx. CMV antigens were checked weekly up to three months, and monthly thereafter. At least one measure of positive CMV antigen (CMV Ag^+^) (with some duplication of the patients) was included in the modeling. CMV infection was considered a binary variable (at least one episode vs. none). The recipient’s sex, age, body mass index (BMI in kg/m²), and the underlying disease at LTx were included in the analyses. All of the recipients were categorized into three main groups in terms of their pre-transplantation underlying disease: bronchiectasis, chronic obstructive pulmonary disease (COPD), and pulmonary fibrosis (as the reference group). 

Statistical Analysis

When an outcome variable such as a biomarker, is measured repeatedly during follow-up, the occurrence of a fatal event can cause significant missing data for the biomarker. In this case, there are two endpoints: time-to-event outcome, which is time to an event of interest, and longitudinal outcome, which is observed over time. Applying a two-stage model and time-dependent Cox model data may lead to biased estimations if the survival outcome is affected by the longitudinal trajectory [8, 10]. Joint modeling can be used to provide valid and efficient inferences [11], evaluate effect of prognostic factors on both endpoints simultaneously [12], to adjust inferences about longitudinal data containing outcome-dependent non-ignorable missing data [13], and to characterize the relationship between longitudinal observations and time-to-event of interest. There are different approaches to fit joint modeling [11-13]. This study used shared random effects for two sub-models. A linear mixed effect model for *j*^th^ longitudinal measure for the *i*^th^ recipient was as follows: 

$Y_{\mathrm{ij}}=\beta_{0l}+\beta_{1l}t_{\mathrm{ij}}I_{t_{\mathrm{ij}\epsilon \left(0,\mathrm{10}\right]}}+\beta_{2l}t_{\mathrm{ij}}^{2}I_{t_{\mathrm{ij}\epsilon \left(0,\mathrm{10}\right]}}+\beta_{3l}t_{\mathrm{ij}}I_{t_{\mathrm{ij}\epsilon \left(10,+\infty \right]}}+u_{i}+\epsilon_{\mathrm{ij}}$

(1)

An accelerated failure time Weibull model with a shape parameter p with random effects was considered for the survival time. The corresponding hazard function at time *t*_i_ for the *i*^th^ recipient was as follows: 

$h\left(t_{i}\right)=p\times e^{\sum_{k}\beta_{k}X_{k}+{\mathrm{\alpha u}}_{i}}t^{p-1}$

 (2)

Where u_i_ is a shared random effect following normal distribution and independent of *e*_ij_~N(0, *σ*^2^*I*), *α* is an association parameter that links two sub-models. *X*_k_ is a variable and *β*_k_ is the corresponding effect. In the survival component of the model, the hazard ratio (HR) showing a ratio of the hazard rates corresponding to the conditions described by two levels of an explanatory variable is estimated. HR was calculated as follows [14].

$\mathrm{HR}=e^{-\beta \times p}$

The joint model was fitted to each covariate one-at-a-time and finally significant covariates were retained in the final model. Data were presented as mean±SD for continues variables and frequency (%) for categorical variables. Estimations were reported along with 95% CIs and p values. Data analyses were performed using proc nlmixed in Statistical Analysis System (SAS ver 9.2). All tests were two-tailed. A p value <0.05 was considered statistically significant.

## RESULTS

A total of 38 patients who had been undergone LTx was included in the study. The mean±SD age of recipients at LTx was 36±14.5 years. The mean of follow-up period was 3±1.7 years. Characteristics of the recipients are presented in Table 1; 11 (29%) recipients experienced AR; 20 (53%) recipients had CMV.

**Table 1:** The recipients’ characteristics

Characteristic	Mean±SD or n (%)
Age at transplant (yrs)	36±14.5
Sex	
Male	28 (74)
Female	10 (26)
BMI (kg/m²)	20.2±4.5
AR*	
None	27 (71)
At least once	11 (29)
CMV^†^	
None	18 (47)
At least once	20 (53)
Underlying lung disease	
Pulmonary fibrosis	13 (34)
Bronchiectasis	16 (42)
COPD^‡^	9 (23)

* Acute rejection;

† Cytomegalovirus;

‡ Chronic obstructive pulmonary disease

**Figure 1:**
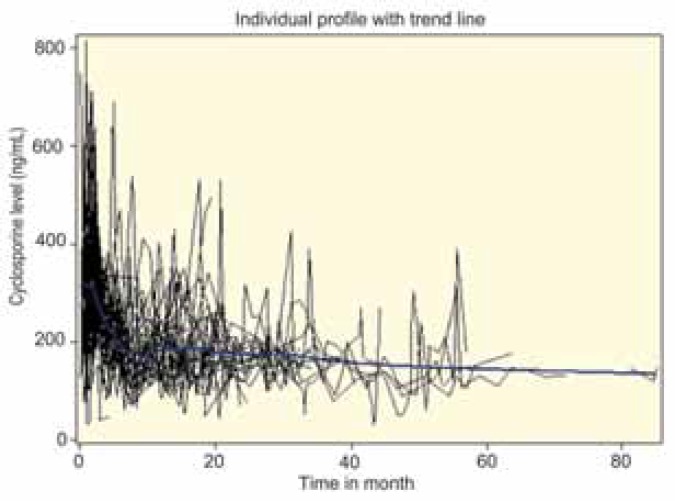
Recipients’ profile plot with average trend line of serum cyclosporine level (ng/mL) over time

A total of 1019 SCL measurements was recorded. The minimum and maximum numbers of SCL measurements were 4 and 64, respectively. The SCL measuring time points were different for each patient. For the recipients who experienced AR, attention was paid to the SCL before and after the rejection. The mean SCL one month before and after AR was 103.5 and 200.3 ng/mL, respectively. The pattern of recipients’ SCL over time in months followed a smooth curve (Figure 1). The fluctuation showed a quadrature form at the first 10 months post-LTx and had a slightly decreasing linear trend for the SCL afterwards.

Univariate joint analysis (not shown) indicated that sex and age were not associated with risk of death (p=0.302, p=0.216, respectively). Recipients with a higher BMI at LTx had a lower mortality risk though the difference was not significant (p=0.891). For the risk of death, no significant difference was found between recipients who categorized into bronchiectasis (p=0.525) and COPD (p=0.403) vs. those with pulmonary fibrosis. AR history was significantly associated with risk of death (p=0.030). CMV infection did not have any significant effects on mortality (p=0.613). The result of the final joint model is shown in Table 2. The risk of mortality for recipients who had at least 1 episode of AR was 1.54 times that of recipients who had none (95% CI: 1.08–2.19). Moreover, the joint model demonstrated that there was a highly significant quadrature form in SCL during the first 10 months post-LTx. A significant linear decreasing trend in SCL after 10 months—2% per month in average (95% CI: -0.03 to -0.019) fited the data very well (p<0.001). 

**Table 2:** The results of joint modeling of time-to-event data and cyclosporine levels

Survival component of the model		
Term in the model	HR^†^ (95% CI)	p value
Intercept	0.16 (0.07 to 0.32)	<0.001
AR* (at least once *vs*. none)	1.54 (1.08 to 2.19)	0.017
Serum cyclosporine level (SCL)		
Term in the model	Estimate (95% CI)	p value
Intercept	0.36 (0.21 to 0.52)	<0.001
${\mathrm{Time}}_{I_{t_{Y}\epsilon (0,10]}}$	0.13 (0.04 to 0.21)	0.002
${{\mathrm{Time}}^{2}}_{I_{t_{Y}\epsilon (0,10]}}$	0.02 (0.03 to 0.01)	<0.001
${\mathrm{Time}}_{I_{t_{Y}\epsilon (10+\infty ]}}$	0.02 (0.03 to 0.019)	<0.001
Association parameter		
*α*	0.8 (0.81 to 0.78)	0.001

* Acute rejection,

† Hazard ratio

Table 2 shows that the association parameter estimate (α = -0.8) was negative and significant, demonstrating strong evidence of an association between the sub-models (Eq 1) and (Eq 2) (p=0.001). This shows that a higher level of SCL was associated with lower risk of death. In addition, the significant variance of α (not shown) confirmed significant heterogeneity among recipients (p=0.038).

## DISCUSSION

Previous reports showed a low survival rate and median life after LTx in Iran [3, 15] and associated risk factors with survival in Iranian recipients [3]. This study, not only evaluated the association between SCL pattern and mortality, but also studied significant determinants of mortality. A new and precise joint model took into account the time-dependent measurements of SCL. Recently, joint modeling has been used frequently to evaluate the associations between longitudinal and time-to-event data such as CD4 count and survival in AIDS studies [16]; urine measurements and acute kidney injury after surgery [17]; and IgG antibody titer and recurrence of pemphigus [18]. 

Calcineurin immunosuppression regimens have used sequential protocols, to reduce the incidence of early calcineurin-associated nephrotoxicity, decrease the incidence and duration of delayed graft function, and decrease the early AR incidence [19]. The significant effect of SCL in solid organ transplantations has previously been reported [5] although scare studies have been conducted on LTx. This study revealed that a higher SCL was significantly associated with a lower mortality risk. Moreover, this study found that the mean SCL decreased over the study period.

A number of studies have shown that age is related to mortality. According to the ISHLT registry, in which 830 recipients received transplants because of idiopathic pulmonary fibrosis, age is related to early (90-day) mortality [20]. In 2012, a study revealed that older age at the time of transplantation can predict increased risk of death significantly (HR=1.03) [21]. Another research reported a significant association between age greater than 55 years post-LTx with an increased risk of mortality an transplantation day, and one and five years post-transplantation [22]. This study showed that age was not associated with mortality based on our findings. Although the effect of age was not significant, the effect direction was compatible with the global findings implying that the older the age, the higher the mortality risk. 

Despite these studies, not all research has confirmed an effect of sex. In 2012, an investigation on 174 patients found that female recipients had lower survival rates [21], supporting the conclusions of a similar study on 240 patients [23]. Similar result was found in other studies [23-25]. However, a study including 332 patients found a non-significant effect of sex on mortality within 90 days post-LTx [24]. It was found no significant effect of sex on mortality rate post-LTx.

The effect of BMI was also investigated. According to the Institute of Medicine (IOM) guideline, underweight is defined as a BMI <18.5 kg/m^2^; normal weight, BMI of 18.5–24.9 kg/m^2^; overweight, BMI of 25–29.9 kg/m^2^; and obesity, BMI ≥30 kg/m^2^. Mortality was shown to be higher in underweight, overweight, and obese patients than those with normal weight in 11,411 recipients in United Network for Organ Sharing data [26]. Moreover, patients with a BMI <17 kg/m^2^ or >25 kg/m^2^ had a higher risk of mortality within 90 days post-transplant [23]. In a nationwide cohort including 5978 recipients using Cox model, it was shown that both underweight and obese recipients had a higher mortality, controlling for the underlying disease. It was concluded that healthy weight for patients with lung disease should be considered a long time before transplantation [27]. In the current sample, no significant association was found between BMI and mortality rate. Nevertheless, almost 60% of studied patients were underweight at the time of LTx, which may explain the non-significance effect of BMI on mortality. 

Underlying diseases other than emphysema were predictive of decreased survival (HR=6.5) [21]. In a single-center study, a multivariable Cox regression model demonstrated no statistical significant relationship between mortality within first year and the pre-transplant diagnosis [25]. The findings in another research [23] showed that the disease category was not a risk factor of death during the first three months after LTx. The present findings showed that the underlying disease was not associated with a higher risk of death. 

The ISHLT report in 2011 revealed that acute rejection affects the risk of death [2]. Based on our findings, AR was a significant risk factor associated with mortality. Unlike the findings of Edwards, *et al* (2011), CMV infection was not a significant risk factor for mortality among Iranian LTx recipients. The non-significant effect of some of the factors might be due to the low number of recipients. Therefore, a multicenter study including more recipients might differentiate the effect of the factors more accurately.

In summary, AR was found to be a risk factor for mortality in Iranian LTx recipients. Although SCL should be monitored regularly after LTx, given the association found between the SCL and mortality in this study, it is recommended to pay serious attention to changes in SCL in the overall survival assessment in Iranian LTx recipients in this center. Though joint modeling is an observation-oriented approach, i.e., the actual sample size of this study was 1019, the small number of recipients was the main limitation of this study that limited generalization of the results. These findings could be a jumping off point for future multicenter studies with higher sample sizes. In addition, Bayesian analysis might be used to overcome the small sample size limitation.

## References

[1] Vadnerkar A, Toyoda Y, Crespo M (2011). Age-specific complications among lung transplant recipients 60 years and older. J Heart Lung Transplant.

[2] Christie JD, Edwards LB, Kucheryavaya AY (2011). The Registry of the International Society for Heart and Lung Transplantation: twenty-eighth adult lung and heart-lung transplant report—2011. J Heart Lung Transplant.

[3] Hosseini-Baharanchi FS, Hajizadeh E, Najafizadeh K (2014). Risk factors associated with survival after lung transplant in Iran. Exp Clin Transplant.

[4] Flechner SM, Kobashigawa J, Klintmalm G (2008). Calcineurin inhibitor-sparing regimens in solid organ transplantation: focus on improving renal function and nephrotoxicity. Clin Transplant.

[5] Citterio F ( 2004). Evolution of the therapeutic drug monitoring of cyclosporine. Transplant Proc.

[6] Nemati E, Einollahi B, Taheri S (2007). Cyclosporine trough (C0) and 2-hour postdose (C2) levels: which one is a predictor of graft loss?. Transplant Proc.

[7] Chaparro-Mutis C, Hutcheon M, Singer L KS ( 2007). Lung transplantation manual.

[8] Wu L, Liu W, Yi GY, Huang Y (2011). Analysis of longitudinal and survival data: joint modeling, inference methods, and issues. Journal of Probability and Statistics.

[9] Stewart S, Fishbein MC, Snell GI (2007). Revision of the 1996 working formulation for the standardization of nomenclature in the diagnosis of lung rejection. J Heart Lung Transplant.

[10] Tsiatis AA, Davidian M (2004). Joint modeling of longitudinal and time-to-event data: an overview. Stat Sin.

[11] Guo X, Carlin BP (2004). Separate and joint modeling of longitudinal and event time data using standard computer packages. Am Stat.

[12] Henderson R, Diggle P, Dobson A (2000). Joint modelling of longitudinal measurements and event time data. Biostatistics.

[13] Hogan JW, Roy J, Korkontzelou C (2004). Tutorial in biostatistics. Handling drop-out in longitudinal studies. Stat Med.

[14] Kleinbaum D, Klein M (2005). Survival analysis. A self-learning approach.

[15] Abbasi A, Sheykh K, Daneshvar AG (2010). Survival of lung transplantation patients in Masih Daneshnavi Hospital. Iranian Journal of Surgery.

[16] De Gruttola V, Tu XM (1994). Modelling progression of CD4-lymphocyte count and its relationship to survival time. Biometrics.

[17] Khodayari-Moez E (2013). The joint analysis of longitudinal and time-to-event data and its application on medical data.

[18] Malehi AS, Hajizadeh E, Ahmadi K, Mansouri P (2014). Assessing the autoantibody levels in relation to recurrence of pemphigus: Joint modeling of longitudinal measurements and recurrent event times. Iran Red Crescent Med J.

[19] Cherikh WS, Kauffman H, McBride MA (2003). Association of the type of induction immunosuppression with posttransplant lymphoproliferative disorder, graft survival, and patient survival after primary kidney transplantation1. Transplantation.

[20] Whelan T, Dunitz J, Kelly R (2005). Effect of preoperative pulmonary artery pressure on early survival after lung transplantation for idiopathic pulmonary fibrosis. J Heart Lung Transplant.

[21] Jacques F, El-Hamamsy I, Fortier A (2012). Acute renal failure following lung transplantation: risk factors, mortality, and long-term consequences. Eur J Cardiothorac Surg.

[22] George TJ, Arnaoutakis GJ, Beaty CA (2012). Acute kidney injury increases mortality after lung transplantation. Ann Thorac Surg.

[23] Madill J, Gutierrez C, Grossman J (2001). Nutritional assessment of the lung transplant patient: body mass index as a predictor of 90–day mortality following transplantation. J Heart Lung Transplant.

[24] Hackman K, Snell G, Bach L (2013). Diabetes Dramatically Decreases Survival in Lung Transplant Recipients. J Heart Lung Transplant.

[25] Osho AA, Castleberry AW, Snyder LD (2014). Assessment of different threshold preoperative glomerular filtration rates as markers of outcomes in lung transplantation. Ann Thorac Surg.

[26] Allen JG, Arnaoutakis GJ, Weiss ES (2010). The impact of recipient body mass index on survival after lung transplantation. J Heart Lung Transplant.

[27] Lederer DJ, Wilt JS, D’Ovidio F (2009). Obesity and underweight are associated with an increased risk of death after lung transplantation. Am J Respir Crit Care Med.

